# Recent high-severity wildfires in a dry-conifer landscape are unprecedented over five centuries and foretell future forest loss

**DOI:** 10.1073/pnas.2513731123

**Published:** 2026-08-31

**Authors:** Andreas P. Wion, Jens T. Stevens, Jonathan D. Coop, Calvin A. Farris, Sean A. Parks, Craig D. Allen, Ellis Q. Margolis

**Affiliations:** ^a^ U.S. Geological Survey, Fort Collins Science Center, New Mexico Landscapes Field Station, Santa Fe, NM 87508; ^b^ School of Environmental and Forest Sciences, University of Washington, Seattle, WA 98195; ^c^ Clark School of Environment and Sustainability, Western Colorado University, Gunnison, CO 81231; ^d^ National Park Service, Division of Fire and Aviation Management, Pacific Southwest Region, Klamath Falls, OR 97601; ^e^Ariel Re UK Limited, London EC3R 8AF, United Kingdom; ^f^ W.A. Franke College of Forestry and Conservation, University of Montana, Missoula, MT 59812; ^g^ Department of Geography and Environmental Studies, University of New Mexico, Albuquerque, NM 87106

**Keywords:** fire severity, fire regime change, neutral landscape model, Jemez Mountains, tree-ring fire-scar

## Abstract

Dry forests of the western United States evolved with frequent, spatially extensive, low-severity fires, followed by a century of fire exclusion. Recently, these same forests have experienced large and increasingly severe fires causing widespread forest loss. To what extent do modern high-severity fires depart from pre-1900 fire regimes, and how do these departures affect the future persistence of dry-conifer forests? We address these questions using a multiscalar approach that combines the largest landscape-scale tree-ring fire-scar network in North America with neutral landscape model simulations in the Jemez Mountains of northern New Mexico. Fire scars recorded a continuous 400-y history (ca. 1500–1900 CE) of frequent, low-severity fires across individual sites, landscapes, and centuries. We evaluated whether forests exhibiting this record could plausibly persist if they had historically burned under modern patterns of high-severity fire. Across the 240,000-ha Jemez landscape, high-severity fires from 1995–2024 burned 32% of fire-scar sites and 25% of dry-conifer forests. Within the Las Conchas Fire perimeter—a 61,000 ha area reburned by multiple fires—66% of fire-scar sites and 71% of forests were burned at high severity, erasing multiple centuries of forest and fire history. Simulations indicate that the probability of the observed fire-scar network persisting under modern high-severity fire rates is near-zero (*P* < 0.001). Projecting these dynamics forward indicates rapid loss of both dry-conifer forests and tree-ring fire histories—50% of modern dry-conifer forest will remain by 2055, and 24% by 2115. Modern high-severity fire regimes appear incompatible with dry-conifer forest persistence.

## Significance

Across a large dry-conifer forest landscape, the area burned by high-severity fire in the past 30 y is unprecedented in five centuries. Historically, frequent low-severity fires were a stabilizing force, but this fire regime collapsed by 1900 due to land use changes and fire exclusion. Since 1995, dry-conifer forest losses resulting from uncharacteristic high-severity fires and minimal postfire regeneration have eroded the ecological and cultural legacies of historical low-severity fire and conifer forests from this historically fire-adapted landscape. If recent rates of large, high-severity fires continue, half of modern dry-conifer forests will remain by 2055 in the Jemez Mountains. Restoring widespread low-severity fire, a historically stabilizing process, can increase resilience in dry-conifer forest landscapes.

Frequent, low-severity surface fires were an important stabilizing ecological process for centuries in diverse forests across North America (1, 2). These fires maintained forest structure and composition while also promoting ecosystem resilience to future fire (3–5). Across the western United States, low-severity fire regimes collapsed by *circa* 1900 through the combined effects of active fire suppression, landscape-scale livestock grazing that consumed fine fuels, and the erasure of Indigenous fire stewardship—collectively referred to as fire exclusion (1, 6, 7). This resulted in a widespread fire deficit that has persisted in many forests despite recent increases in area burned (8, 9). Over the past several decades, climate change and fuel accumulation have driven a sharp increase in the extent and severity of wildfires (2, 10–12), with far-reaching consequences for human communities, carbon dynamics, and biodiversity (13–15). While the shift toward larger high-severity fires (i.e., killing most or all trees) in recent decades is well documented (12, 16, 17), the long-term context of such shifts are only beginning to be understood (18–20). Is the extent of modern high-severity fire (1995–2024) in dry-conifer forests uncharacteristic compared to historical fire regimes (i.e., 1500–1900)? If so, what do these changes in fire regimes portend for the future of forests in these landscapes?

Tree-ring fire scars provide annually resolved and spatially explicit records of low-severity surface fires that repeatedly wounded but did not kill trees (21). The abundance of such fire-scar records on surviving trees documents the absence of high-severity fire at these same point locations for centuries. Such records, compiled in the North American tree-ring fire-scar network (NAFSN), offer multicentury reconstructions of historical fire frequency, severity, and extent, particularly in well-sampled regions like the southwestern United States (2, 6, 18). The Jemez Mountains of northern New Mexico are the most densely sampled landscape in North America, containing over 1,200 fire-scarred trees sampled from 110 sites within a ~240,000 ha forested landscape. Tree-ring fire-scars and charcoal sediment records in the Jemez Mountains (22–25) chronicle multiple centuries of recurring surface fires. In ponderosa pine and mixed-conifer forests (hereafter dry-conifer forests), fires regularly removed dead fuel and killed small-diameter and fire-intolerant trees, fostering low-density and open-canopy forests with grassy understories (2, 4, 22–25). These frequent fires from both abundant lightning and Indigenous burning (6) reduced fire severity and created heterogeneous age and size structures across the Jemez Mountains landscape, favoring large, fire-resistant, old-growth trees (4).

**Fig. 1.**
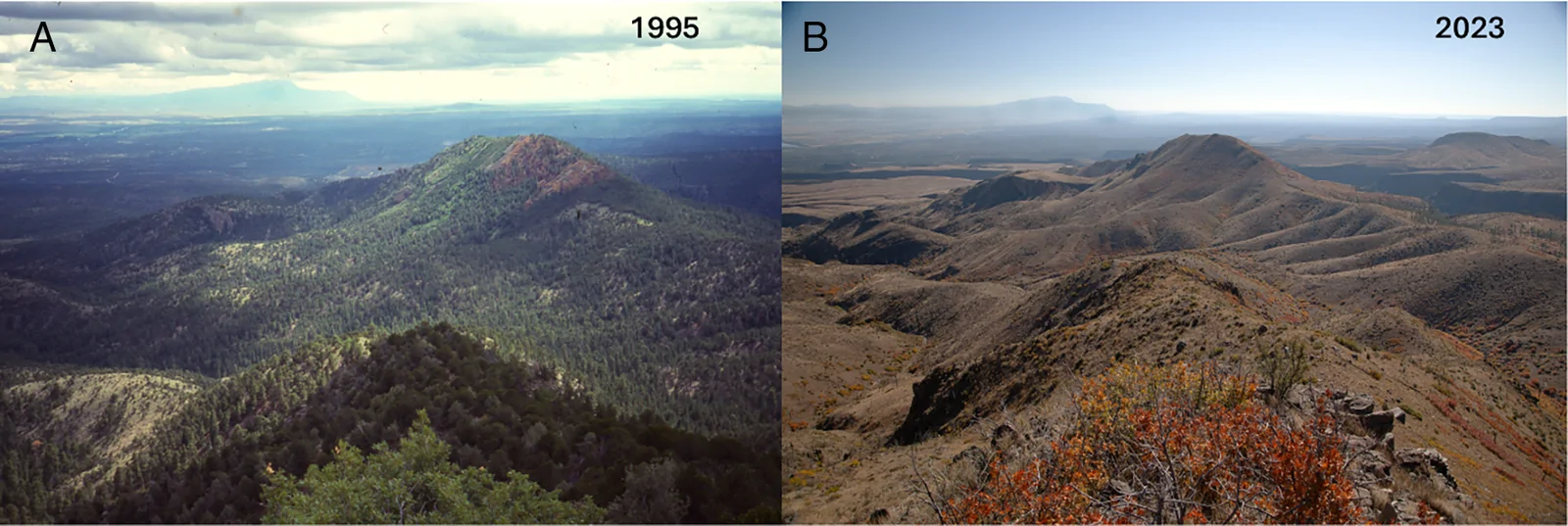
Dry-conifer forest loss from high-severity fires in the Jemez Mountains, New Mexico (1995–2023). (*A*) Photograph of dry-conifer forest from St. Peter’s Dome Lookout, facing south. (*B*) Repeat photograph showing the near-complete conifer forest loss (mortality and lack of regeneration) following two high-severity fires (1996 Dome Fire and 2011 Las Conchas Fire). Multiple tree-ring fire-scar sites with a history of frequent, low-severity fire are located within and just outside of this field of view. Photo credits: C.D. Allen 1995 and 2023.

The Jemez Mountains landscape also prominently illustrates the rapid fire-catalyzed transformations underway in many fire-adapted conifer forests of western North America (15) (Fig. 1). Since 1995, tens of thousands of hectares (ha) of conifer forests in the Jemez Mountains have burned at high severity, subsequently converting extensive landscapes to shrubland and grassland ecosystems devoid of tree regeneration (14, 26). Fire-catalyzed forest loss in this system stems from a collapsed surface-fire regime and a warming climate, leading to alternate, stable, shrub-dominated states that are unlikely to recover to a prefire state (15, 27, 28). Patterns of shrubfield establishment in the Jemez Mountains have been linked to severe droughts, fire, and Indigenous burning practices (7, 28, 29); yet the maximum size (368 ha) and cumulative area of pre-1995 shrub patches in the Jemez Mountains (29) are quite small relative to the shrubfields observed emerging here postfire since 1995 (Fig. 1). In Southwest US dry-conifer forests, high-severity fire triggers persistent deforestation when contiguous patches of tree mortality exceed the dispersal capacity of surviving conifer seed trees (15). In addition, subsequent reburns (14), increasing climate aridity (20), and competition from resprouting shrubs and grasses (26, 29), all strongly constrain postfire conifer regeneration. As high-severity patches expand, their interiors can become increasingly disconnected from seed sources and overtaken by resprouting species, remaining nonforest for decades to centuries or longer (15, 28, 29).

The largest modern fire in the Jemez Mountains was the 2011 Las Conchas Fire, which burned 61,056 ha. This fire reburned at least three previous modern fires (Dome Fire—1996, Oso Complex Fire—1998, Cerro Grande Fire 2000). In 2022, the Cerro Pelado Fire reburned 15,769 ha of this same area. Large areas of high-severity fire appear incompatible with historical fire regimes and dry-conifer forest persistence, yet the full scope of this departure remains uncertain. To what extent do modern high-severity fires depart from pre-1900 fire regimes, and how do these departures affect the future persistence of dry-conifer forests? Answering such questions can inform conservation of dry-conifer forests across the western United States. To contextualize modern fire severity in the Jemez Mountains across time and space, we:

- Characterized the fire history of dry-conifer forests across the Jemez Mountains using the local tree-ring fire-scar network (1);
- Quantified the rate of forest and fire-scar loss from recent high-severity fires (1995–2024);
- Modeled the likelihood that the fire-scar network could have persisted under simulated past configurations of high-severity fire patches comparable in size to those seen today at multiple spatial scales; and
- Simulated the trajectory of continued forest loss from high-severity fire into the future across spatial scales.

Fire histories of the Jemez Mountains were described using the median fire return interval derived from 110 fire-scar sites across this dry-conifer forest landscape. Sites consisted of groups of fire-scarred trees in small plots (median area = 2 ha). Historical (pre-1900) large fire years were identified as those in which at least 10% of sites recorded fire scars. To characterize the modern fire regime, high-resolution postfire aerial imagery was used to characterize the size and distribution of high-severity fire patches—areas that were recently conifer forests but now are treeless—at two spatial scales: 1) the Las Conchas Fire (61,000 ha) and 2) the Jemez Mountains (240,000 ha). Configurations of modern high-severity fire patches were then simulated using neutral landscape models (NLMs) and classified into patches using quantile-based thresholds. To test for the departure of modern fire severity from the historical fire regime represented by the fire-scar network, we estimated the likelihood that any configuration of spatially randomized modern high-severity patches would preserve the fire-scar network intact (i.e., would not burn the fire-scar sites at high severity). To project the future extent of forest loss from high-severity fire, simulated high-severity patch configurations were successively overlaid onto the landscape using the observed contemporary rates of high-severity fire at 30-y intervals, calculating the remaining forest cover at each step. The results provide a plausible range of forest trajectories into the near future based on observed rates of recent high-severity fire.

## 2. Results

### 2.1. Fire History.

**Fig. 2.**
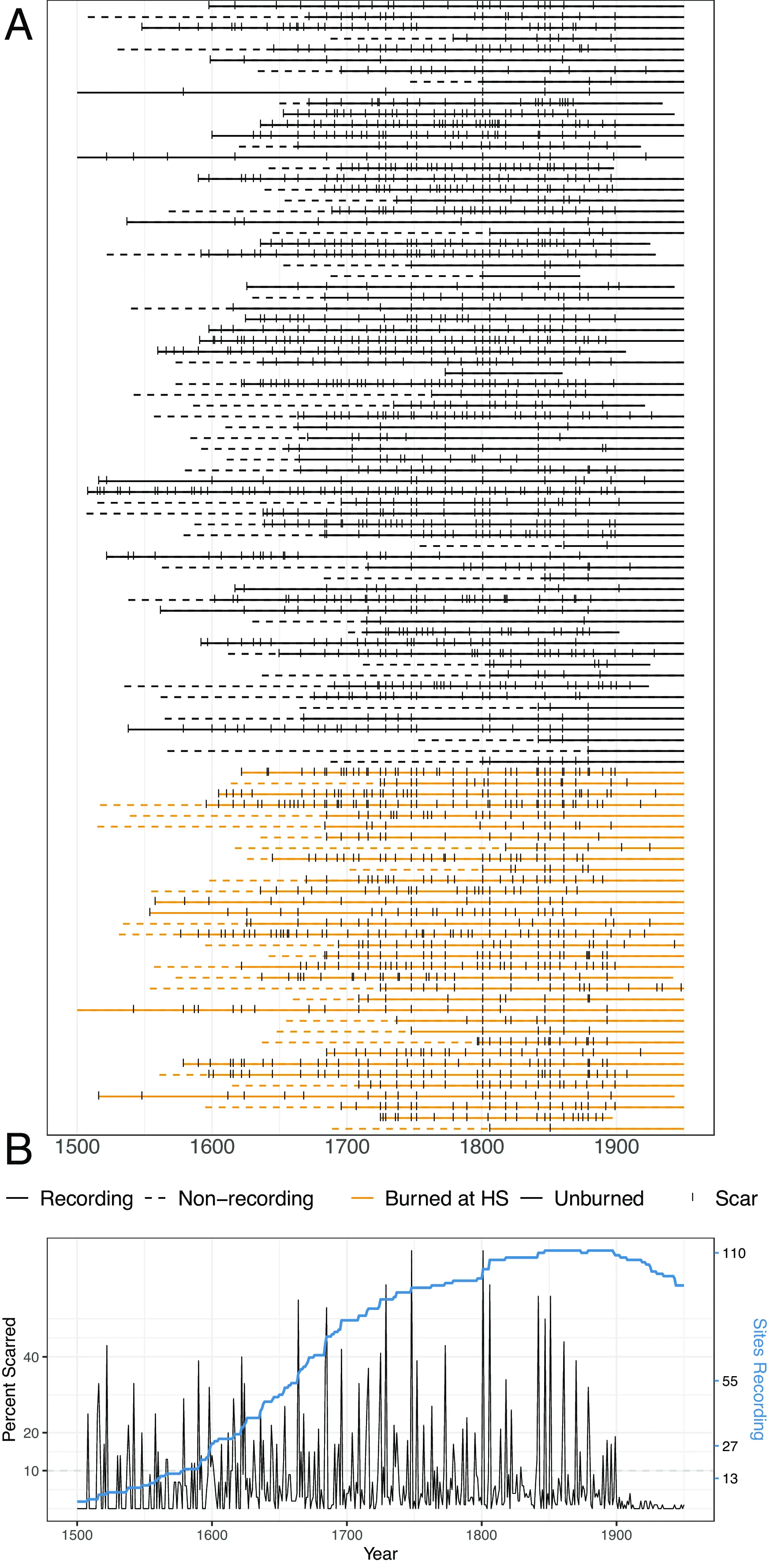
Tree-ring reconstructed fire history in the dry-conifer forests of the Jemez Mountains, New Mexico (1500–1950). (*A*) Composite fire-scar chronologies of sites with ≥2 recording trees. Sites that have burned at high severity since 1995 are colored in orange (32%). (*B*) The percentage of recording fire-scar sites that burned each year (black line). The blue line is the number of fire-scar sites that are recording by year. Sites are considered recording after the first fire scar is recorded on a tree, which allows subsequent fires to be recorded by the open wound. Data from the North American Fire-Scar Network (NAFSN) (1).

Fire-scar records from the Jemez Mountains indicate that low-severity fires were historically frequent and spatially extensive in dry-conifer forests. Between 1508 (the first recorded fire) and 1899, large fires scarring 10% or more of fire-scar sites occurred every 3 y (*landscape median*). Throughout this period, 102 large fires burned in this landscape, with a maximum interval of 12 y. Median fire intervals were stable over time despite differences in sample depth (Fig. 2). Over 30-y periods, median fire intervals ranged from 1 to 6 y (composite landscape median fire intervals = 3.2, SD = 1.3 y). Notable large fire years occurred in 1748 and 1801 (68% of sites burned), as well as 1729 and 1806 (59% sites burned). The last large fire year was in 1899, when 19% of sites burned. With the onset of fire exclusion, 78 y passed before the next significant fire in the Jemez Mountains (1977 La Mesa Fire, 6,200 ha). The interval between 1899 and 1977 was six times greater than the longest interval between large fires in the prior four centuries. Under pre-1900 fire regimes, 20 large fire years were expected to have occurred from 1900 to 1976, representing a large fire deficit.

### 2.2. Modern Fire Severity.

**Fig. 3.**
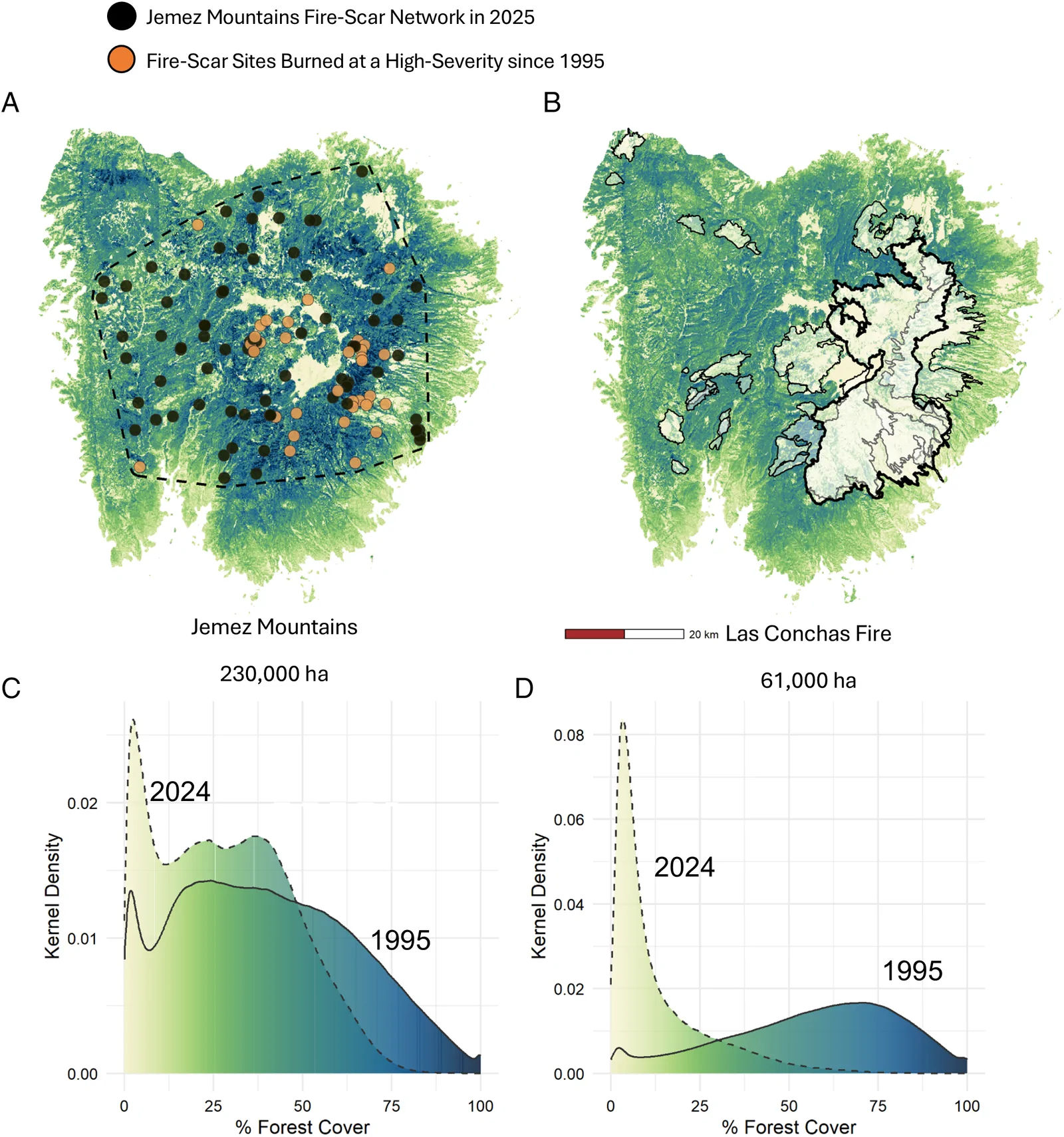
The tree-ring fire-scar network, modern high-severity fires, and changes in forest cover across the Jemez Mountains, New Mexico (1995–2024). (*A*) Forest cover in 1995 (percent; green-blue background, see panel *C* or *D* for legend) and fire-scar sites (orange and black dots) in the Jemez Mountains. The dashed line is a convex hull representing a 1.5 km buffer around all fire-scar sites. (*B*) Forest cover in 2024 overlaid with fire perimeters greater than 404 ha that burned between 1995 and 2024. The 2011 Las Conchas Fire is highlighted as a bold outline. (*C* and *D*) Change in forest cover frequency distributions within the Jemez Mountains (*C*) and the perimeter of the Las Conchas Fire (*D*). The colors of *A* and *B* correspond to the x-axis of panels *C* and *D*. Forest cover maps were downloaded from Rangeland Analysis Platform (30) using Google Earth Engine.

Since 1995, 29 fires have burned over 100,000 ha of the 240,000 ha landscape (Table 1 and Fig. 3). About one-third of the total burned area (34,700 ha) was reburned by two or more fires. Approximately 61,100 ha of this forested landscape, or 25%, has burned at high severity in the last 30 y. The 2011 Las Conchas Fire alone burned 43,500 ha at high severity. During this same 30-y period, 32% of the Jemez Mountains fire-scar network has been burned by high-severity fire (35 sites, Fig. 3). Three-quarters of all burned sites were located within the perimeter of the Las Conchas Fire (25 sites, 66% of the Las Conchas fire-scar network). Each of these sites previously survived and recorded multiple past fires as fire scars over centuries, suggesting that such a loss within three decades is highly uncharacteristic of historical fire regimes.

**Table 1.** Descriptions of modern fire characteristics (1995–2024) across two spatial scales analyzed in the Jemez Mountains, New Mexico

Spatial extent	Extent (ha)	Area burned (ha)	Area burned at high severity (ha)	Number of fire-scar sites burned at high severity	Total fire-scar sites
Jemez Mountains	240,200	103,376	61,107 (25.4%)	35 (31.8%)	110
Las Conchas Fire	61,056	61,056	43,510 (71.2%)	25 (65.8%)	38

High-severity was defined as fire-driven forest loss observed from 0.6m^2^ resolution aerial imagery and fire-scar data were compiled from the NAFSN (1).

### 2.3. Are Modern Fires Burning with Uncharacteristic Severity?

**Fig. 4.**
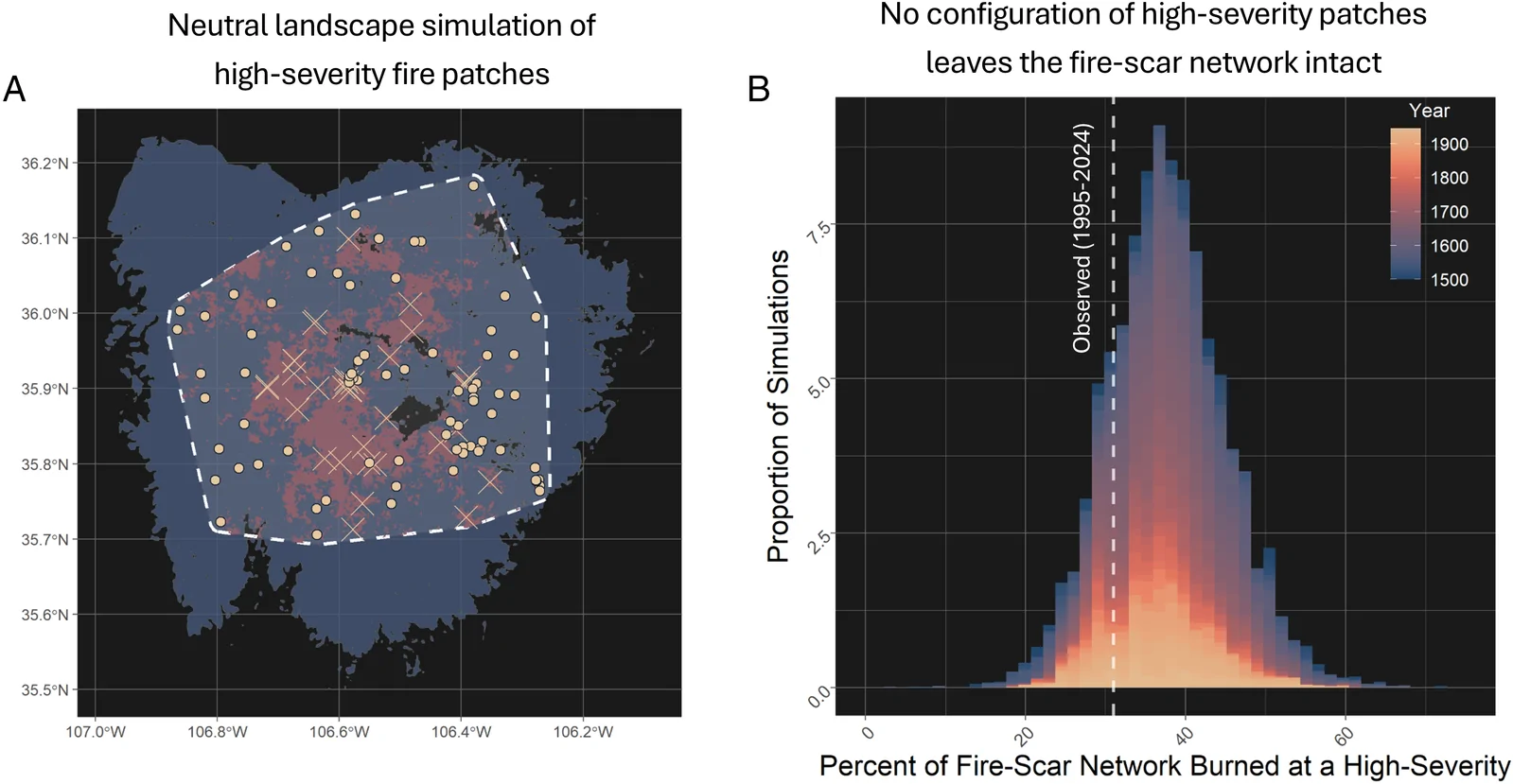
Simulated fire-scar network loss from high-severity fire using neutral landscape models (NLMs) in the Jemez Mountains, New Mexico. (*A*) An example NLM simulation, representing a single iteration of randomized modern high-severity patches (red shapes) and their intersection with fire-scar sites (intersecting—yellow X, nonintersecting—yellow dot). The map of the Jemez Mountains (dark blue) and the fire-scar core area (dashed line) are displayed for reference. Bootstrapped replicates are combined to generate probability distributions observed in *B*. (*B*) Histogram representing the frequency of the percentage of fire-scar sites burned by simulated high-severity fire over 30-y periods within the sampled fire-scar network. Changes in fire-scar sample depth through the addition or loss of a site over time are analyzed by calculating the intersection between the existing network and one hundred random samples of NLM simulations in 30-y moving windows from 1500 to 1950. The dashed white line denotes the observed proportion (32%) of fire-scar sites burned at a high severity in the Jemez Mountains from 1995 to 2024.

No arrangement of simulated modern high-severity fire patches preserved the observed fire-scar network intact (Fig. 4). Therefore, the probability that the fire-scar network could persist through fires of comparable high-severity extent in the historical past was effectively zero (*P* < 0.001). Bootstrap simulations of 500-y histories indicated that modern high-severity fire extents would consistently eliminate large portions of the fire-scar network in any 30-y window. The distribution of outcomes was centered well above zero site loss - more than 99% of simulations eliminated at least 20% of the fire-scar network (Fig. 4*B*). Simulated high-severity fires burned an average of 37.7% of fire-scar sites (95% CI, 23 to 53%) within any 30-y window. Given that all simulations burned a large fraction of the fire-scar network, these simulated alternative histories could not have produced the observed fire-scar record. This indicates modern high-severity fires have no analog within the 500-y history of documented fire.

### 2.4. Future Trajectories of Forest Loss.

**Fig. 5.**
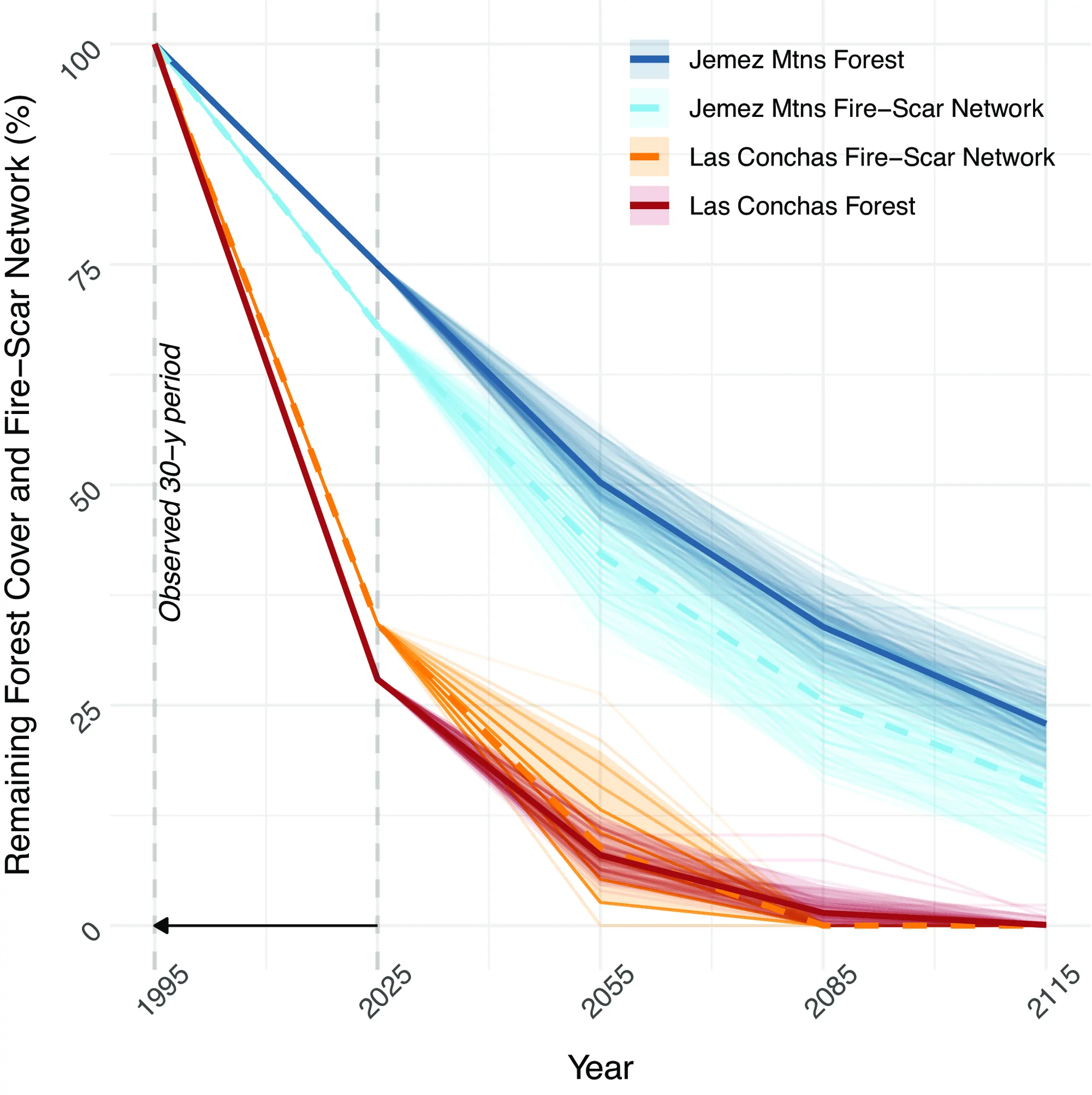
Observed and projected forest cover and fire-scar sites remaining after multiple simulated fires at two spatial scales in the Jemez Mountains (1995–2115). Projections are based on observed rates of modern high-severity fire from 1995 to 2024. Dashed lines denote the mean trajectory of the fire-scar network, and solid lines indicate the mean trajectory of forest cover. Individual faint lines represent unique simulation trajectories, and ribbons represent the 95% range of the simulations. Simulations are constrained to either the 61,000-ha extent of the 2011 Las Conchas Fire, or to the 240,000-ha extent of Jemez Mountains. Simulations were run for three 30-y periods (2025 – 2055, 2056 – 2085, 2086 – 2115).

Future trajectories of forest loss were simulated at two spatial scales—the Jemez Mountains and the Las Conchas Fire footprint. In the Jemez Mountains, simulations indicated that less than a quarter of the pre-1995 Jemez Mountains forest cover (24%, 95% CI, 19 to 30%) and the fire-scar network (16%, 95% CI, 8% to 25%) will remain by 2115, given the current rate and extent of high-severity fires within this landscape (Fig. 5). Within the Las Conchas Fire perimeter (Fig. 3, bold outline) forest cover declines to 10% of pre-1995 levels (95% CI, 4% to 12%) by 2055 and near zero by 2085 (1%, 95% CI, 1% to 5%). Fire-scar sites in Las Conchas follow a similar decline, with 8% remaining by 2055 (95% CI, 1% to 18%) and none are predicted to remain by 2085 (Fig. 5).

## 3. Discussion

### 3.1. Modern High-Severity Fires Have No Analog Over 500 Years in the Jemez Mountains.

Through integration of landscape-level simulations and tree-ring fire history, our results demonstrate that modern high-severity fires are without precedent since 1500 in this New Mexico dry-conifer forest landscape. Across all simulated configurations, no arrangement of modern high-severity fire patches could have burned an equivalent area at any three-decade interval in the past 500 years while preserving the observed fire-scar record or the centuries-old forests that produced it. This conclusion holds across multiple spatial and temporal scales, from a large fire footprint to the broader mountain landscape, and from decades to centuries of repeated burning. The Jemez Mountains fire-scar network documents a continuous, long-term regime of frequent, spatially extensive, low-severity fires that persisted across heterogeneous topography, climate variability, and human land use. When modern high-severity fire patches are imposed on this historical template, conifer forests are rapidly erased, along with the fire-scar record itself. This divergence is most clearly illustrated within the 61,000 ha footprint of the Las Conchas Fire—where repeated high-severity fire since 1995 has eliminated 66% of fire-scar sites and 71% of dry-conifer forest cover. The erasure of forest structure and ecological memory has triggered cascading ecological, hydrologic, and social consequences across the Jemez Mountains (27). Similar patterns of fire-catalyzed forest loss are occurring across many historically frequent-fire forests of the western United States (2, 15, 18, 31), indicating that the processes documented here are not unique to the Jemez Mountains. They reflect a broad shift toward fire regimes that exceed the historical capacity of dry-conifer forests to persist and recover.

### 3.2. Consequences of Forest Loss from High-Severity Fire.

Dry-conifer forests evolved over millennia in the presence of frequent, low-severity surface fires and exhibit strong trait-based resistance to fire, including thick bark, elevated crown bases, and fire-tolerant regeneration strategies (32). These traits confer resilience to repeated surface fire but provide limited protection against large, contiguous patches of high-severity fire. As a result, modern fire regimes increasingly exceed the capacity of dry-conifer forests to persist or recover, pushing many landscapes toward alternative, nonforest states (27, 29, 33). Across the southwestern United States, former old-growth dry-conifer forests are being replaced by resprouting angiosperm shrublands and grasses (26), with limited conifer reestablishment following large high-severity fires (28). In the Jemez Mountains, three decades of modern high-severity fire have fundamentally altered forest composition, structure, function, and persistence at landscape scales. This represents an immense departure from historical patterns and foretells future dry-conifer forest loss.

The consequences of these transitions extend beyond vegetation change. In the Jemez Mountains, modern high-severity fires have driven severe watershed responses, including flooding and erosion (34), debris flows (35), and extreme degradation of water quality (36). These effects threaten surface water supplies under an ongoing megadrought (37, 38), release large quantities of carbon sequestered over centuries (33), and erode the cultural connections and access to formerly forested landscapes (39, 40). In combination, these effects represent a rapid loss of ecosystem functions that historically conferred stability and resilience to both human and ecological systems. However, high-severity fire is not uncharacteristic across all western forest ecosystems, nor does it universally lead to forest loss. Stand-replacing fire has historically occurred in high-elevation spruce–fir forests, wetter mixed-conifer forests with aspen, serotinous systems such as lodgepole pine (*Pinus contorta*), and some persistent piñon–juniper woodlands, where species are adapted to recover following high-severity fire under suitable climatic conditions (41–44). Even within the Jemez Mountains, dry-mixed-conifer forests historically experienced limited pockets of mixed-severity fire, including localized tree torching and small stand-replacing patches. However, these historical patches were spatially constrained—generally <300 ha—and occurred within a broader matrix of surface-fire-dominated regimes (22–24, 29). By contrast, modern high-severity fires are occurring at spatial scales that overwhelm historical resilience mechanisms, marking a regime shift in fire effects beyond the historical range of variability (4, 15, 19, 20, 22, 29).

### 3.3. Old Wood and Tree-Ring Fire-Scars Are a Rapidly Dwindling Resource.

Ancient trees and old wood, including fire-scarred trees, are ecocultural witnesses to centuries of fire, climate variability, and human land use. Fire scars record the physical imprint of repeated low-severity fire with annual to subannual precision, preserving spatial and temporal information about fire frequency, extent, season, and synchrony that cannot be reconstructed from any other archive (45–48). These records have been foundational for defining historical fire regimes, understanding forest dynamics, identifying climate–fire relationships, and informing contemporary forest and fire management. The loss of fire-scarred trees and remnant old wood in recent high-severity fires severs our ability to reconstruct past fire regimes, understand past Indigenous fire-use, test emerging hypotheses, or place contemporary fires within a historical context (7, 45–47).

Although more than 2,500 fire-scar collections have been identified and synthesized within the NAFSN (1), large geographic gaps remain, and many existing records now face the risk of loss. At the same time, tree-ring science is rapidly expanding in scope, with new methods using remnant wood to address questions spanning fire ecology, forest dynamics, paleoclimate, and human–environment interactions (7, 48–51). Losing these forests and their associated archives foreclose future discovery. Continued systematic dendroecological sampling—particularly in regions not yet represented—can help preserve these records while they still exist.

### 3.4. Conclusions and Implications.

Modern high-severity fires in dry-conifer forests of the Jemez Mountains have no historical analog in the past five centuries. Evaluated across space and time, contemporary fire severity is incompatible with the persistence of the forests and fire-scar networks that defined pre-1900 fire regimes. If current trends continue, only a quarter of pre-1995 dry-conifer forest cover is likely to remain by 2115 in the Jemez Mountains. High-severity fire erodes dry-conifer forest persistence. Preventing further loss of old-growth forests and their ecological memory can benefit from proactive stewardship that limits high-severity fire. Restoring widespread low-severity fire, a historically stabilizing process, can increase resilience in dry-conifer forest landscapes.

## 4. Materials and Methods

### 4.1. Natural History.

The Jemez Mountains are bounded by the Rocky Mountains to the north and east, the Chihuahuan Desert to the south, and the Colorado Plateau to the west. Elevation ranges from 1,550 m at the intersection of the Jemez River and the Rio Grande—to over 3,500 m at Chicoma Mountain, along the rim of the central caldera. These mountains are volcanic features formed by a series of massive eruptions over the past several million years. The region is underlain by rhyolitic tuff, with deeply incised canyons and mesa tops. The climate is continental and semiarid with a bimodal precipitation pattern—winter snows and summer monsoon rains are separated by pronounced spring drought, with abundant lightning during the warmer months (52). Coniferous vegetation varies with elevation and aspect—from piñon-juniper woodlands of *Pinus edulis* and *Juniperus monosperma* and *Juniperus deppeana* at lower treeline, to dry-mixed-conifer forests consisting of *Pinus ponderosa* var. *scopulorum* and *Pinus strobiformus, Pseudotsuga menziesii,* and *Abies concolor* on the mid-slopes, and smaller pockets of blue spruce (*Picea pungens*), Engelmann spruce (*Picea engelmannii),* and corkbark fir (*Abies lasiocarpa* var. *arizonica)* at the highest elevations and within drainages.

### 4.2. Cultural History.

People have lived within and around the Jemez Mountains for thousands of years (7, 39). These mountains provided water, wood, and fire-adapted ecosystems that supported hunting, gathering, and cultivation. Many Pueblos are currently located around the base of the Jemez Mountains, and this landscape is sacred to numerous Indigenous peoples. The Spanish were the first colonial Europeans to arrive in this region during the late 1500s, bringing with them nonnative flora, fauna, and pastoral livestock traditions (4, 7, 39, 52). This landscape became part of Mexico following independence from Spain in 1821, before being annexed into the United States in 1848. These changes brought additional waves of new settlers, traditions, and land use changes. The arrival of the transcontinental railroad to New Mexico in 1879 brought millions of sheep and cattle that consumed fine fuels across the region and contributed to early fire cessation in this region (4, 40, 53). Forest infilling occurred throughout most of the 20th century as frequent fire ignitions from both lightning and cultural practice were actively suppressed, excluding fire from the Jemez landscape. Following commercial scale logging of fire-resistant trees, younger and smaller sized fuels began to accumulate and become abundant. This forest structure change and fuel accumulation are primary drivers of the severe fires we are seeing amid the backdrop of a 21^st^ century megadrought (2, 5, 15, 18).

### 4.3. Tree-Ring Fire-Scar Network (1500–1950).

Tree-ring fire-scar samples in the NAFSN (1) were collected using a spatially systematic approach combined with opportunistic sampling where fire-scarred material was conspicuous and accessible. Sites extended across a 240,000-ha area within the Jemez Mountains, which broadly corresponds to the distribution of dry-mixed-conifer forests. Sites were required to have >10% tree cover in 1995, excluding a total of six sites from the NAFSN database (1). 110 fire-scar sites (n = 1,240 fire-scarred trees) located within Jemez Mountains were ultimately identified for inclusion in this analysis. A spatial buffer was applied to each centroid site location corresponding to the reported area sampled. If no area sampled was reported, a 0.1 ha radius buffer was applied to the site. Sites contained an average of 11 trees sampled across a median area of 2 ha. Over 99% of these sites contained multiple replicated fire-scars (i.e., crossdated fire-scars across three or more trees). Fire scars were obtained from major forest species present in the Jemez landscape (*SI Appendix*, Appendix 1).

Median fire return intervals (e.g., MFI) were calculated for each unique site (≥2 trees) using the burnr package (54). A two-pass filter was applied to each chronology, removing those fires which scarred fewer than either 10% or 2 recording trees, representing “large” fires. Trees begin recording after they develop the first cross-dated fire scar. Fires do not scar every tree, and not every tree records each fire. Fire scars without adequate replication to meet this threshold were excluded from the analysis. A landscape-level MFI (i.e., a Jemez-wide composite) was derived from all chronologies with an identical 10% and two-site minimum filter.

### 4.4. Contemporary Fire History and High-Severity Burn Severity Mapping (1995–2024).

A total of 29 fires (>404 ha, 1,000 acres) have burned since 1995, including uncontrolled wildfires, wildfires managed for resource benefit, and prescribed fires (Monitoring Trends in Burn Severity (55) [MTBS]. High-severity fire patches were identified using 0.6 m^2^ resolution aerial imagery (USDA National Agricultural Imagery Program [NAIP], 2022) within the large fire perimeters identified through MTBS. A classification approach utilized 3 × 3 pixel focal window texture, variance, and a normalized greenness index coupled with a global canopy height map (56) to identify tree canopy pixels. Rasters were aggregated from 0.6 to 30 m^2^ (0.81 ha^2^), constraining the resolution of all analyses to those large high-severity patches which are likely to exceed native conifer seed dispersal limitations. Seeds of most mixed conifer species are relatively heavy and dispersed by wind and gravity (57, 58), uncommonly traveling further than 100 m beyond a surviving parent tree. While some tree regeneration can exist beyond this distance (59), the rate of forest recruitment relative to shrub and grass establishment strongly indicates that most areas are unlikely to recover to a prefire state within human lifetimes (29, 57), unless located beneath or adjacent to a surviving seed source (28, 60). High-severity patches identified from NAIP corresponded to pixels with less than 2% tree cover derived from aerial imagery. Individual patches were delineated using a queen’s case rule (i.e., 8 possible directions from a focal cell).

### 4.5. Simulating High-Severity Patches.

We used NLMs to simulate patches of high-severity fire. NLMs are spatial representations of a Gaussian distribution and can be used to simulate spatially autocorrelated phenomena, like disturbance. Matern covariance structures provided an excellent fit using a semivariogram (R^2^ = 0.96, Kappa = 0.3), which was then used to derive estimates of the range, sill, and nugget of high-severity patch sizes (a binary field, i.e., has or has not burned at high severity). A mixture of two NLM fields were used to simulate short-range and long-range patch dynamics (small and large patches, respectively). We then iteratively tuned the model mixtures and model parameters until the number and size distribution of simulated patches closely matched the observed distribution of high-severity patches (*SI Appendix*, Fig. S1). 1,000 NLM simulations were generated then projected and masked to the extent of fire-scar sites in the Jemez Mountains. Those simulations within 15% of the target (observed modern) area burned at high severity were retained (606—Jemez Mountains, 106—Las Conchas).

The fading record problem is inherent to paleoecological proxies like fire-scars, as fewer samples occur further back in time due to decay and destruction. To account for the change in sample depth over time in the simulations, a sliding window was applied to the fire scar network—subsetting only those sites recording during a given 30-y period (to match the 1995–2024 observational period). At each change in sample depth during the historical period (1500–1950), a random sample of 100 of these configurations were drawn. Any instance of an intersection between a simulated high-severity fire patch and a recording fire scar site were tallied, deriving a probability distribution of the expected number of fire scarred sites burned given randomized high-severity patch configurations. An empirical cumulative distribution function was used to calculate the probability (i.e., *P*-value) that the fire-scar record would remain intact (i.e., observing zero fire-scar sites burned by high-severity fire) under similar configurations of high-severity fire in the historic past.

### 4.6. Projecting Future Loss of Forests and Fire-Scar Sites.

Last, we used the NLM simulations (above) to project future rates and extents of the loss of forest cover and fire-scar sites. High-severity fire patches were simulated at two spatial scales—the Jemez Mountains and the Las Conchas Fire perimeter. The Las Conchas Fire perimeter included a collection of five large overlapping fires with a significant component of high severity. Individual simulations were overlaid onto maps of contemporary forest cover and the proportion of prefire forest cover pixels was calculated at each step. We used the same approach to estimate loss of future fire-scar sites. This approach is likened to mice incrementally eating a block of cheese—how many holes (high-severity fire patches) can be taken out of the cheese (forest cover) before none is left?

## Supplementary Material

Appendix 01 (PDF)

## Data Availability

Tabular and Spatial data have been deposited in Dryad (https://doi.org/10.5061/dryad.jsxksn0nc) (61).
