# Supplementary material for: Recent high-severity wildfires in a dry-conifer landscape are unprecedented over five centuries and foretell future forest loss
Source: Proc Natl Acad Sci U S A. 2026 Aug 31;123(36):e2513731123. doi: 10.1073/pnas.2513731123 (PMC13552868; doi:10.1073/pnas.2513731123)
Supplement: Supplementary file 1 [file pnas.2513731123.sapp.pdf]

Supporting Information:

Recent high-severity wildfires in a dry-conifer landscape are unprecedented over five centuries and foretell future forest loss

Andreas P. Wion[1]*, Jens T. Stevens[2], Jonathan D. Coop[3], Calvin A. Farris[4], Sean A. Parks[5,6], Craig D. Allen[7], Ellis Q. Margolis[1]

Affiliations and Addresses

1. U.S. Geological Survey, Fort Collins Science Center, New Mexico Landscapes Field Station, 301 Dinosaur Trail, Santa Fe, NM, 87508

2. University of Washington, School of Environmental and Forest Sciences, Seattle WA 98195

3. Clark School of Environment and Sustainability, Western Colorado University, Gunnison, CO

4. National Park Service, Division of Fire and Aviation Management, Pacific Southwest Region, Klamath Falls, OR.

5. Ariel Re UK Limited, London, UK

6. W.A. Franke College of Forestry and Conservation, University of Montana, Missoula, MT

7. Department of Geography & Environmental Studies, University of New Mexico, Albuquerque, NM

*Andreas P. Wion corresponding author

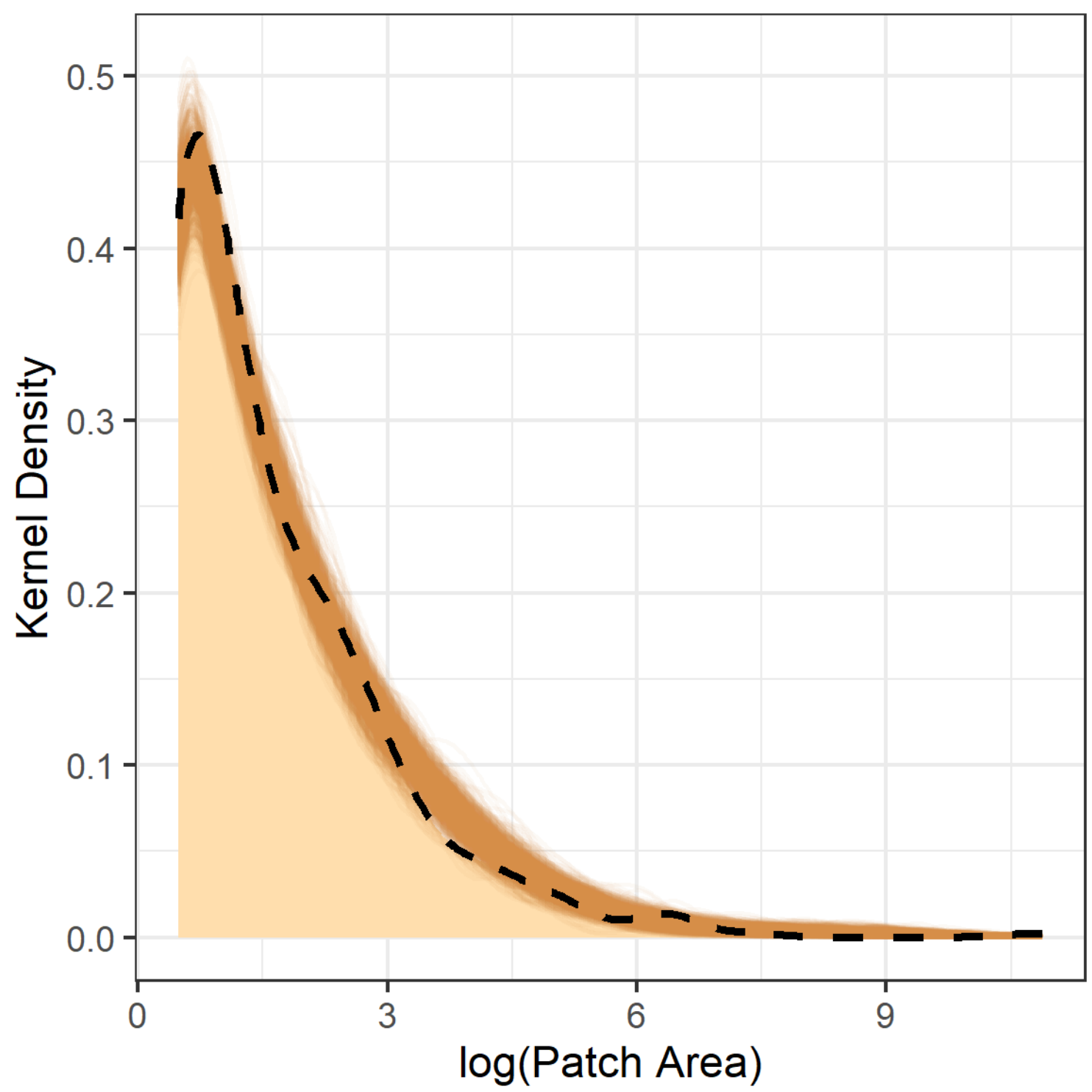

Supplemental Figure 1) The frequency-distribution of individual high-severity fire patch areas (x-axis units are log-transformed meters^2) greater than 0.81 ha^2 in size for the observed fires in the Jemez Mountains (1995-2024, black dashed line). This demonstrates the ability of the neutral landscape simulations to replicate observed high-severity patches (solid dark orange lines).

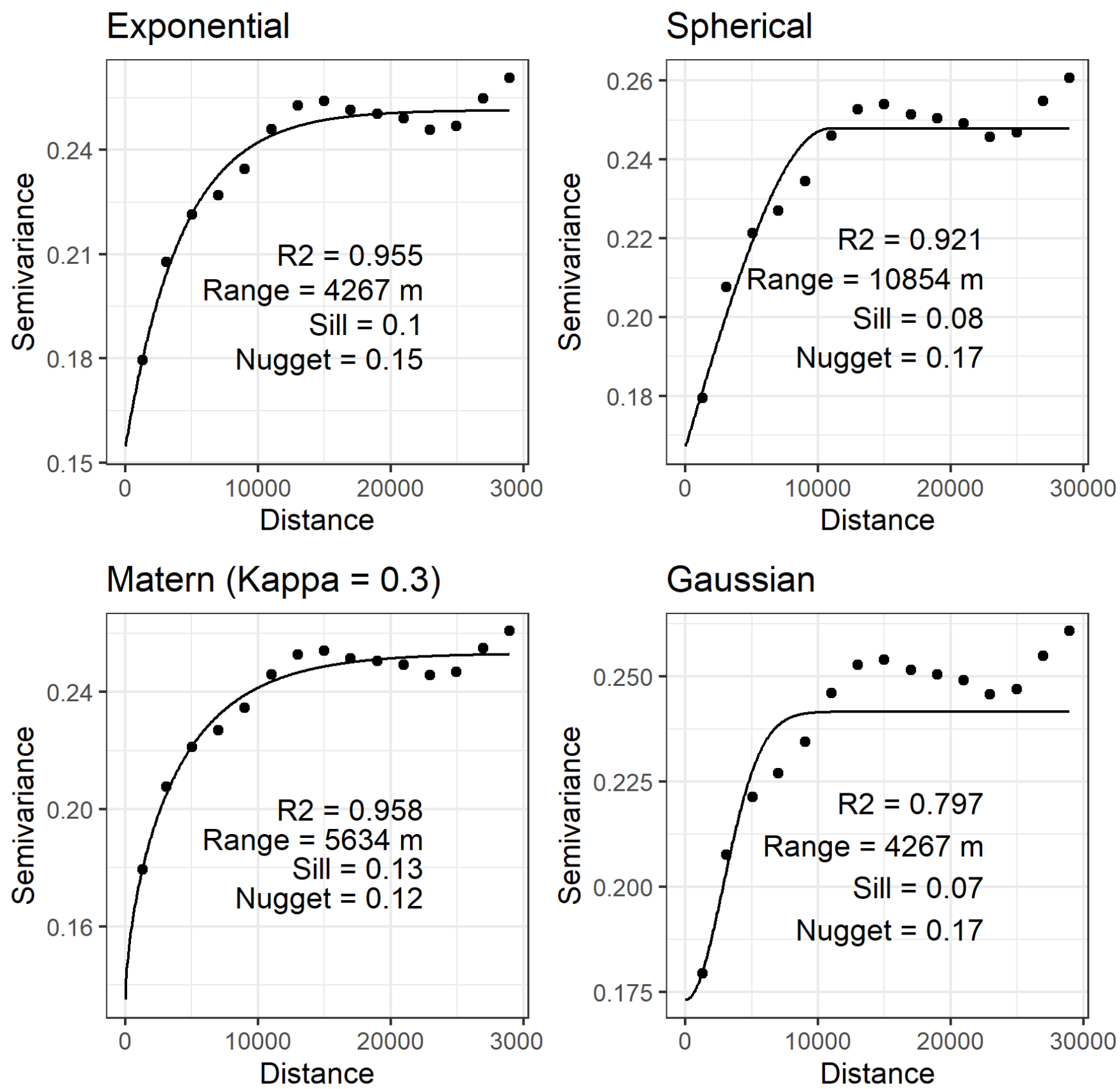

Supplemental Figure 2) Empirical and fitted semivariograms used to derive estimates of parameters when fitting neutral landscape models for high-severity fire simulations. Points represent the empirical and lines represent various fits using different decay functions (Exponential, Matern, Spherical, and Gaussian). A Matern covariance structure outperformed all examined variogram models. Distance units are in meters.

Recent high-severity wildfires are unprecedented over five centuries in a dry-forest landscape

Andreas P. Wion, Jens T. Stevens, Jonathan D. Coop, Calvin A. Farris, Sean A. Parks, Craig D. Allen, Ellis Q. Margolis

Proceedings of the National Academy of Sciences (2026).

Appendix 1) Metadata table of fire-scar sites obtained from the North American Fire-Scar Network in the Jemez Mountains. The first year is the earliest inner tree-ring from a site, and the first scar denotes when a site begins recording fire. The last year is the latest outer tree-ring at the site, and the last scar is the last recorded fire at the site. Tree species abbreviations are as follows: PIPO – *Pinus ponderosa;* PSME – *Pseudotsuga menzezeii*; PISF – *Pinus strobiformus*; ABCO- *Abies concolor*; POTR – *Populus tremuloides*; PCEN – *Picea engelmanii*; QUGA – *Quercus gambelii*; JUSC – *Juniperus scopulorum*.

ID	site_code	Longitude	Latitude	first_year	first_scar	last_year	last_scar	n_trees	Tree species	Area sampled (ha)
Allen_ABH1	ABH1	-106.593	35.90175	1688	1748	1970	1879	4	PIPO	1
Allen_ABH2	ABH2	-106.578	35.908	1567	1716	2002	1879	2	PSME, PISF	1
Allen_ABH3	ABH3	-106.57	35.91123	1689	1716	1995	1851	3	PSME	1
Allen_ABL	ABL	-106.578	35.91386	1753	1789	2002	1879	3	PIPO, PSME	0.5
Allen_ALA1	ALA1	-106.589	35.91274	1463	1503	1996	1879	9	PIPO	1
Allen_ALA2	ALA2	-106.585	35.91181	1565	1624	1996	1879	5	PIPO, PISF	0.5
Allen_ALA3	ALA3	-106.584	35.90857	1665	1716	2002	1879	5	PSME	1

Allen_ALA4	ALA4	-106.588	35.90839	1480	1516	1943	1896	8	PIPO, PISF	0.5
Allen_AM1	AM1	-106.37	35.82064	1615	1684	1973	1899	3	PIPO	0.5
Allen_AM2	AM2	-106.385	35.82331	1561	1585	1987	1908	6	PIPO, PISF, PSME	9
Allen_AM3	AM3	-106.397	35.82256	1459	1480	1987	1893	6	PIPO	4.5
Allen_BAL	BAL	-106.392	35.72909	1450	1532	1995	1940	32	PIPO, PSME	26
Allen_BAN	BAN	-106.336	35.8183	1637	1725	1977	1893	10	PIPO	40
Allen_BHQ	BHQ	-106.273	35.77893	1712	1776	1925	1902	2	PIPO	1
Allen_BJC	BJC	-106.279	35.79464	1612	1650	1987	1957	8	PIPO	43
Allen_CAN	CAN	-106.387	35.91433	1655	1685	1996	1914	32	PSME, ABCO, PISF	4.5
Allen_CAS	CAS	-106.375	35.90692	1378	1480	1993	1966	31	PIPO, PISF	4.5
Allen_CHB	CHB	-106.507	36.04699	1401	1454	2002	1902	13	PIPO, PISF	5
Allen_CME	CME	-106.381	35.89954	1660	1709	1993	1880	6	PIPO	0.5
Allen_CMN	CMN	-106.396	35.90542	1683	1729	1993	1880	20	PSME, ABCO	3
Allen_CMN2	CMN2	-106.393	35.9054	1563	1716	1996	1910	10	PSME, ABCO	7
Allen_CPE	CPE	-106.38	36.17	1380	1522	1993	1959	26	PIPO	15.5

Allen_CPM	CPM	-106.353	35.77567	1554	1624	1990	1955	15	PIPO	25
Allen_CPU	CPU	-106.413	35.79104	1573	1637	1942	1893	8	PIPO	1.5
Allen_CSE	CSE	-106.405	35.85082	1579	1622	1987	1899	4	PIPO, PSME	2
Allen_CSW	CSW	-106.418	35.85628	1587	1631	1988	1899	6	PIPO	8
Allen_ESC	ESC	-106.365	35.83014	1557	1599	1994	1899	11	PIPO, PSME	5.5
Allen_FR1	FR1	-106.372	35.81689	1642	1679	1987	1893	3	PIPO	0.5
Allen_FR2	FR2	-106.397	35.81378	1595	1604	1987	1943	3	PIPO	1.5
Allen_FR3	FR3	-106.408	35.81883	1580	1600	1987	1899	5	PIPO	1
Allen_FRM1	FRM1	-106.271	35.77051	1611	1638	1996	1902	18	PIPO, PIED	8.5
Allen_FRM2	FRM2	-106.272	35.76399	1592	1605	1996	1892	8	PIPO, PIED	5
Allen_FWH	FWH	-106.277	35.77735	1584	1664	1958	1867	17	PIPO, PIED	18
Allen_FWH1	FWH1	-106.278	35.77842	1610	1664	1955	1869	30	PIPO, PIED	1
Allen_LOG	LOG	-106.541	35.79855	1534	1573	1991	1925	13	PIPO, PSME, ABCO	1.5
Allen_MCN01	MCN_01	-106.624	35.80669	1408	1493	1972	1909	30	PIPO	115
Allen_PIPE	PIPE	-106.338	35.89329	1598	1654	1997	1929	27	PIPO	9

Allen_PME	PME	-106.38	35.88838	1702	1773	1993	1962	16	PSME, ABCO, POTR	45.5
Allen_PMR	PMR	-106.38	35.88372	1626	1632	1993	1912	23	PIPO, PSME, PISF	5.5
Allen_PMW	PMW	-106.405	35.89701	1617	1669	1993	1925	10	PSME, PCEN	1
Allen_POL	POL	-106.466	36.09588	1407	1537	1996	1879	2	PISF	1
Allen_SAW	SAW	-106.434	35.82819	1654	1725	1987	1873	3	PIPO	0.5
Allen_SECO	SECO	-106.477	36.09575	1422	1480	1997	1922	15	PIPO, PISF	1.5
Allen_UEF	UEF	-106.408	35.8442	1530	1542	2000	1899	9	PIPO	2
Allen_UWF	UWF	-106.425	35.83851	1539	1562	1987	1861	5	PIPO, PISF	11
Allen_VIL	VIL	-106.312	35.89164	1410	1538	1998	1883	11	PIPO, PISF	3.5
Baisa_ABR	ABR	-106.493	35.92556	1240	1248	1897	1896	6	PIPO	NA
Baisa_ABU	ABU	-106.583	35.91671	1595	1654	2002	1899	6	PIPO	NA
Baisa_VGR	VGR	-106.501	35.88099	1636	1654	1967	1900	11	PIPO	1.5
Carri_SDP	SDP	-106.599	35.80205	1642	1652	1898	1887	2	PIPO	1
Dewar_VEC	VEC	-106.561	35.83753	1640	1650	1997	1904	11	PIPO	67
Dewar_VG	VG	-106.469	35.87966	1418	1530	2005	1992	95	PIPO, PSME	4955
Dewar_VJ	VJ	-106.523	35.91849	1517	1573	2005	1992	42	PIPO	815

Dewar_VSA	VSA	-106.556	35.96991	1526	1542	2005	1969	45	PIPO, PISF, PSME	2260
Dewar_VSC	VSC	-106.569	35.93694	1484	1586	2005	1929	18	PIPO	195
Dewar_VSLD	VSLD	-106.559	35.94461	1614	1684	1960	1908	11	PIPO	225
Dewar_VSR	VSR	-106.518	35.94213	1479	1501	2005	1971	21	PIPO, PSME	468
Dewar_VTW	VTW	-106.481	35.97594	1660	1748	1965	1870	1	PIPO	358
Farel_SAYS	SAYS	-106.637	35.74019	1639	1678	1991	1977	9	PIPO	15
Farel_TOV	TOV	-106.765	35.79428	1650	1668	1934	1868	12	PIPO	7.5
Guite_MPB	MPB	-106.687	36.08939	1302	1473	2013	2010	15	PIPO, QUGA, JUSC	340
Guite_PGM	PGM	-106.795	35.72287	1555	1625	2013	1993	9	PIPO, QUGA	32
Guite_RDC	RDC	-106.591	35.87604	1345	1467	2014	2013	25	PIPO, QUGA, PISF	76
Guite_SNN	SNN	-106.861	36.00373	1170	1250	2013	1899	26	PIPO, QUGA	292
Guite_SNS	SNS	-106.866	35.97852	1376	1480	2013	1991	13	PIPO, QUGA	243
Guite_SPC	SPC	-106.847	35.9664	1596	1714	2014	1989	20	PIPO, QUGA	191
Margo_APW	APW	-106.773	36.02522	1562	1578	1966	1873	10	PIPO	2

Margo_BAC	BAC	-106.798	35.82014	1535	1583	1924	1910	11	PIPO	1
Margo_BLC	BLC	-106.744	35.97209	1371	1587	1976	1870	12	PIPO	1
Margo_BQC	BQC	-106.535	36.09947	1630	1715	1982	1876	4	PSME	2
Margo_BRC	BRC	-106.674	35.92017	1448	1471	2016	1879	10	PIPO, PISF	7
Margo_BST	BST	-106.514	35.74711	1648	1664	2017	2011	8	PIPO, PSME	1
Margo_CPS	CPS	-106.357	35.94429	1754	1806	2017	2000	5	PISF, PIPO, PSME	1
Margo_CYB	CYB	-106.634	36.10963	1507	1622	1961	1870	11	PIPO, PISF, PSME	4
Margo_CYC	CYC	-106.603	36.05346	1515	1576	1950	1902	8	PIPO	3
Margo_ELF	ELF	-106.669	35.87216	1388	1401	2016	1971	10	PISF, PIPO	1
Margo_HME	HME	-106.733	35.79928	1557	1576	1989	1926	13	PIPO, PSME	5
Margo_JCE	JCE	-106.803	35.77859	1586	1712	1921	1890	9	PIPO, PISF	1.5
Margo_JPE	JPE	-106.278	35.99536	1542	1632	2017	2000	10	PIPO	0.5
Margo_LFC	LFC	-106.756	35.85304	1573	1616	1995	1898	11	PIPO	2
Margo_LGR 1	LGR1	-106.552	35.80068	1700	1752	1993	1861	2	PSME, PIPO	1
Margo_LGR 2	LGR2	-106.551	35.8012	1411	1423	1860	1806	6	PSME, PIPO	1

Margo_LHV	LHV	-106.313	35.9452	1573	1631	2017	2000	10	PISF, PIPO	1
Margo_LNL	LNL	-106.35	35.86655	1463	1474	1907	1890	13	PIPO, PISF	2
Margo_MLN	MLN	-106.585	36.09876	1371	1479	2016	1870	11	PIPO	3
Margo_MPC	MPC	-106.562	35.74726	1478	1605	2009	1899	10	PIPO, PISF	1
Margo_MRI	MRI	-106.484	36.01243	1540	1616	2016	1861	9	PISF, PSME	0.5
Margo_MSC	MSC	-106.351	35.97729	1630	1684	2017	2011	9	PIPO, PISF	0.5
Margo_MSP	MSP	-106.573	36.13217	1119	1150	1943	1902	10	PIPO	1.5
Margo_OAC 2	OAC2	-106.674	35.93696	1688	1786	1873	1847	3	PSME, PIPO	1
Margo_OAC 3	OAC3	-106.674	35.93684	1653	1748	1986	1873	9	PSME, PIPO	1
Margo_OJI	OJI	-106.755	35.92136	1522	1572	1929	1896	11	PIPO	2.5
Margo_PAL	PAL	-106.578	35.71275	1469	1611	1925	1896	11	PIPO	2.5
Margo_PER	PER	-106.507	35.76982	1645	1729	2011	1890	8	PIPO, PSME, PISF	1
Margo_PNE	PNE	-106.503	35.80395	1636	1681	1961	1887	11	PIPO, PSME	0.5
Margo_RBS 1	RBS1	-106.582	35.89751	1573	1789	1847	1842	1	PIPO	1

Margo_RBS3	RBS3	-106.582	35.89792	1515	1654	1991	1896	5	PIPO, ABCO	1
Margo_RPN	RPN	-106.711	36.01386	1568	1654	1968	1899	11	PIPO, PSME	1.5
Margo_SBN	SBN	-106.82	35.88744	1445	1583	1950	1896	10	PIPO	1
Margo_SJC	SJC	-106.622	35.75167	1620	1658	1918	1893	7	PIPO	3.5
Margo_SSP1	SSP1	-106.719	35.90064	1379	1441	1970	1896	12	PIPO	1
Margo_SSP2	SSP2	-106.718	35.90291	1489	1654	1966	1896	14	PISF, PIPO	1
Margo_TCN	TCN	-106.827	35.92024	1459	1637	1943	1870	8	PIPO	0.5
Margo_TWC2	TWC2	-106.642	35.98686	1465	1488	2012	1880	26	PISF, PIPO, PSME, ABCO	1
Margo_TWC3	TWC3	-106.639	35.98902	1747	1801	2011	1896	5	PISF, PCEN, PIPO, PSME	1
Margo_TWS	TWS	-106.684	35.81723	1634	1696	2015	1976	12	PIPO	4
Margo_UCC	UCC	-106.583	36.03738	1074	1125	2016	1860	12	PISF	1
Margo_USA	USA	-106.447	35.94757	1688	1757	2016	2011	11	PIPO	1.5
Margo_UWC	UWC	-106.635	35.90063	1469	1499	2016	1899	12	PISF, PIPO	0.5

Margo_VCW	VCW	-106.646	36.0542	1508	1579	1955	1899	10	PIPO, PISF, PSME	2.5
Morin_NAB	NAB	-106.58	35.92	1412	1422	1996	1860	7	PISF, PIPO	0.5
Swetn_BAHQ	BAHQ	-106.523	35.8608	1637	1716	1989	1888	4	PIPO	0.5
Swetn_BOL	BOL	-106.637	35.70604	1701	1714	1902	1880	21	PIPO	NA
Swetn_CCC	CCC	-106.82	35.9965	1538	1548	1978	1881	20	PIPO	40
Swetn_EFK	EFK	-106.56	35.82273	1407	1483	1999	1899	15	PIPO, PSME	2.5
Swetn_GAM	GAM	-106.328	36.02326	1531	1558	1979	1921	25	PIPO, QUGA	148
